# Impact of Cooling Curve Type on Sperm DNA Integrity During Cryopreservation

**DOI:** 10.5935/1518-0557.20260047

**Published:** 2026

**Authors:** Letica Avi, João Paolo Bilibio, Ana Carolina Martinhago, Martina Cordini, Alfred Paul Senn, Vera Lucia Langaro Amaral

**Affiliations:** 1 Departamento de Reprodução Assistida, FertiBC Centro de Reprodução Assistida, Balneário Camboriú, SC, Brazil; 2 Departamento de Biomedicina, UNIVALI, Itajaí, Brazil

**Keywords:** sperm DNA fragmentation, cooling rate, semen, cryopreservation techniques, reproductive medicine

## Abstract

**Objective:**

To assess whether sperm DNA fragmentation is affected differently by cryopreservation techniques using different cooling curves before immersion in liquid nitrogen (LN₂).

**Methods:**

A paired case-control study was conducted with 21 semen samples from men undergoing fertility evaluation. Each sample (Fresh Group) was initially analyzed for sperm concentration, vitality, motility, and DNA fragmentation. The remaining semen was divided into two groups and subjected to different cryopreservation protocols: 1) preliminary refrigeration for 20 min at a cooling rate of -0.9°C/min, by exposure to liquid nitrogen vapor (Refrigerated Group), 2) direct exposure to liquid nitrogen vapor at a cooling rate of -20.5°C/min (Vapor Group).

**Results:**

Both cooling protocols (Refrigerated and Vapor) caused a significant reduction compared with the Fresh Group in progressive motility (49.0% *vs*. 30.4% and 27.4%, *p<*0.001) and sperm vitality (85.1% *vs*. 64.9% and 64.1%, *p<*0.001). No significant differences were observed between the Refrigerated and Vapor Groups in terms of recovery of vitality and motility (*p=*0.361 and *p=*0.718, respectively). DNA fragmentation increased from 27.4% in the Fresh Group to 39.6% in the Refrigerated Group and to 36% in the vapor Group (*p<*0.001). However, the Vapor Group showed a lower mean DNA fragmentation rate than the Refrigerated Group (*p*=0.001).

**Conclusion:**

Cryopreservation using direct exposure to liquid nitrogen vapor was associated with lower sperm DNA damage compared with the protocol involving pre-refrigeration, suggesting that faster cooling may better preserve DNA integrity during freezing.

## INTRODUCTION

Cryopreservation is a widely used technique for maintaining human sperm viability over extended periods (Huang *et al*., 2022; Tao *et al*., 2020). It involves controlled cooling of semen samples through a defined temperature gradient before immersion in liquid nitrogen (LN₂) (Nijs & Ombelet, 2001). This approach is essential for fertility preservation, particularly in oncology patients, sperm donors, and men with infertility-related conditions such as varicocele, offering an opportunity to preserver fertility for future reproductive use (Oehninger *et al*., 2000).

Various cryopreservation protocols differ in cooling rate, cryoprotectant concentration, and packaging method. Cooling may occur gradually or rapidly before LN₂ exposure, and alternative methods such as vitrification enable direct immersion in LN₂ without gradual cooling (O’Neill *et al*., 2019; Tao *et al*., 2020). Although cryopreservation is routinely performed, it still induces cellular damage through oxidative stress, ice crystal formation, dehydration, and osmotic imbalance, which can compromise sperm function and DNA integrity (Nijs & Ombelet, 2001; O’Neill *et al*., 2019).

Despite its widespread use, no universal standard exists for sperm freezing protocols, and differences in cooling curves-such as nitrogen vapor exposure time or prior refrigeration-can significantly affect post-thaw recovery (O’Neill *et al*., 2019; Tao *et al*., 2020; Riva *et al*., 2018). Such variability reduces reproducibility across laboratories (Bandularatne & Bongso, 2002; Muñoz *et al*., 2016). Moreover, it remains unclear how the cooling rate itself influences sperm DNA fragmentation, a factor strongly associated with reduced fertilization potential, impaired blastocyst formation, and increased miscarriage risk (Du & Tuo, 2023; Raad *et al*., 2018).

The integrity of sperm DNA is a critical determinant of reproductive success, as fragmentation reflects the cumulative effects of oxidative stress and cryo-induced injury. Understanding how different cooling dynamics affect this parameter is therefore essential for improving outcomes in assisted reproduction.

This study aims to address this knowledge gap by directly comparing two controlled cooling curves, one including a pre-refrigeration step and another involving direct LN₂ vapor exposure. By using precise temperature monitoring of identical semen samples, this work provides a quantitative assessment of how cooling dynamics influence sperm motility, vitality, and DNA fragmentation, contributing to the optimization of cryopreservation protocols in clinical practice.

## MATERIALS AND METHODS

### Study Design

This was a paired case-control study in which each case served as its own control. A total of 21 seminal samples from men undergoing fertility evaluation at the FertiBC Assisted Reproduction Center (Balneário Camboriú, SC, Brazil) were included. Participants were invited and provided informed consent before inclusion. The inclusion criteria included comprised men meettng the World Health Organization (WHO, 2021) reference values for sperm parameters, specifically with normal sperm concentration, motility, and vitality. These criteria ensured that only samples with adequate sperm quality were used for analysis.

### Ethical Approval

The study protocol was approved by the Research Ethics Committee of the University of Vale do Itajaí (UNIVALI; approval no. 6.993.335). Participation was voluntary and anonymous. All participants provided written informed consent and received their analysis results. The study adhered to the 1975 Helsinki Declaration revised in 2013.

### Sample Collection and Semen Analyses

Semen samples were obtained by masturbation and maintained at 37°C until liquefaction. The interval between semen collection and cryopreservation did not exceed 60 minutes.

Fresh samples were assessed for sperm concentration (Makler chamber, Sefi-Medical Instruments, Israel; 200× magnification), motility (categorized as progressive (a+b), non-progressive (c), or immotile (d); 400x), vitality (eosin 0.5%, Sigma-Aldrich, USA; 400), morphology (Panótico stain, Laborclin, Brazil; 200 spermatozoa, 1000×), and DNA fragmentation (Sperm Chromatin Dispersion Test, Halosperm®, Halotech DNA, Spain; 200-400 spermatozoa; 1000 (Fernández *et al*., 2005)).

### Temperature Monitoring

**Figure 1:**
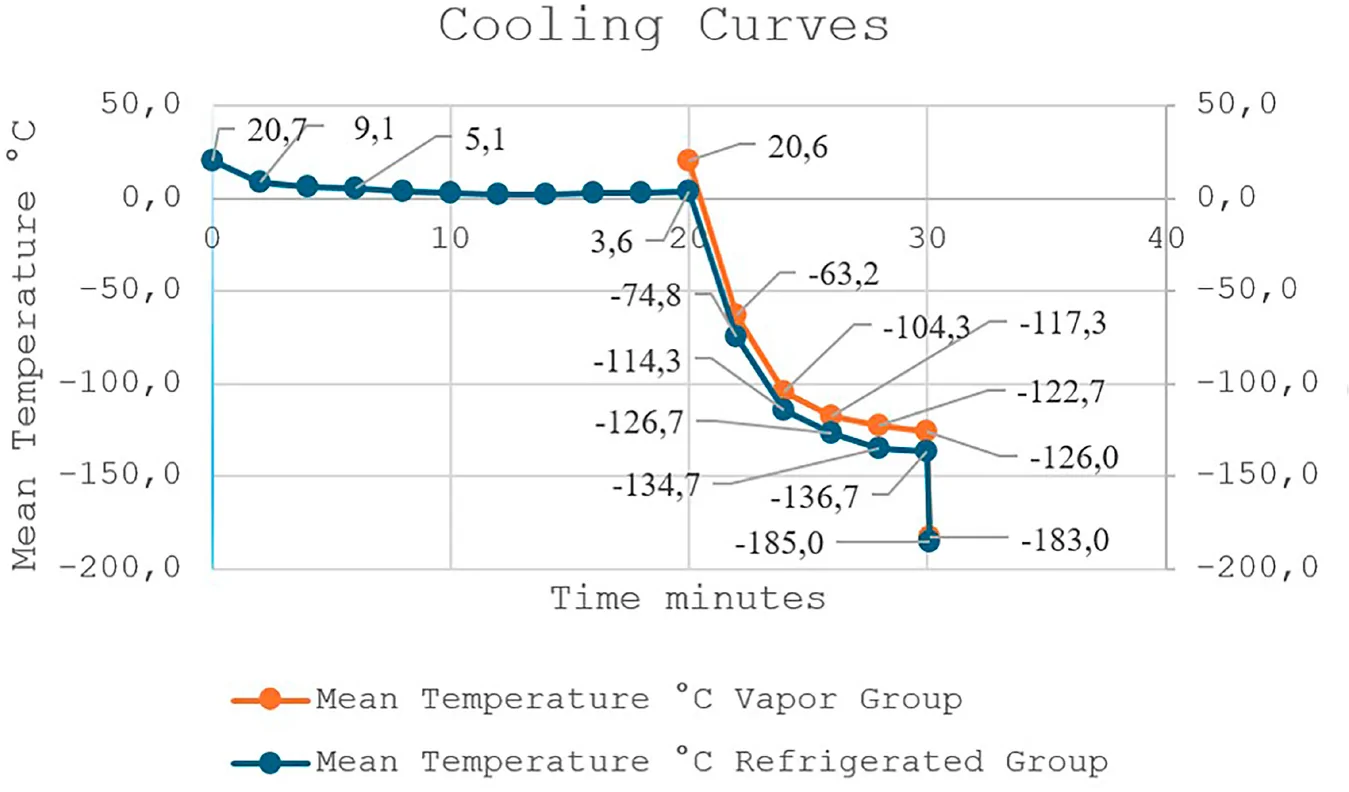
Cooling curves comparing: Temperature monitoring during cryopreservation protocols for Refrigerated Group (blue line, n=21) and Vapor Group (red line n=21).

Sample and ambient temperatures were monitored in triplicate using calibrated thermocouples (Akso^®^, Akso instruments, Brazil) placed inside the semen straws and in the laboratory air, with recordings every 2 minutes. Ambient air remained stable at 20°C, the refrigerator equilibrated at 3°C after 10 minutes, and in nitrogen vapor temperature reached after 10 minutes of exposure was -136°C (Vapor Group) and -126°C (Refrigerated Group) (Figure 1).

### Sample cryopreservation protocols

An aliquot of each fresh semen sample was diluted 1:1 with a cryoprotective medium (Freezing Medium TYB, FujiFilm Irvine Scientific, USA) and filled into 0.5 mL straws. The straws were divided into the two following groups with distinct cryopreservation protocols (Figure 1).

1. **Refrigerated Group:** Pre-refrigeration during 20 min from ambient temperature (20°C) down to refrigerator temperature (2-3°C) at a rate -0.9°C/min, followed by exposure to nitrogen vapor for 10 min and immersion in liquid nitrogen.
2. **Vapor Group:** Direct exposure to N2L vapor at a cooling rate of -20.5°C/min for 10 minutes, followed by immersion in liquid nitrogen.

For the liquid nitrogen vapor stage, the straws were placed on a custom support previously described (Amaral *et al*., 2025). Labelled cryopreserved straws of both groups were then stored in a cryogenic container until processed for post-thaw assessments.

### Thawing and Post-thaw Assessment

All samples were thawed at 37°C for 10 minutes. Post-thaw evaluations included motility, vitality, and DNA fragmentation, according to same procedures described for fresh semen.

### Statistical Analysis

Data were analyzed using SPSS v.25 (IBM Corp., New York, USA**).** Descriptive statistics were presented as means±standard deviations (SD). Two-tailed paired t-tests were conducted to compare pre-freeze and post-thaw values for sperm vitality, motility, and DNA fragmentation. Logistic regression (two-tailed) analysis was additionally performed to assess the strength and direction of associations between cryopreservation protocols and post-thaw sperm outcomes. Regression coefficients (β), odds ratios (OR), and 95% confidence intervals (CI) were calculated for each parameter (DFI, motility, and vitality). Statistical significance was defined as *p*<0.05.

### Power Calculation

A power calculation was performed to assess the statistical power of the study based on the sperm DNA fragmentation results. With an expected mean difference of 3.5% between the Vapor Group (35.9%) and the Refrigerated Group (39.5%), the estimated effect size (Cohen’s d=0.78) indicated that 21 paired samples provided 80% power at α=0.05. The actual post hoc power achieved for detecting this difference was 0.83 (83%), confirming adequate sensitivity of the sample size for the observed effect. The 95% confidence interval for the mean difference in DNA fragmentation between groups was 1.8-5.2%.

## RESULTS

A total of 21 participants were included in the study, with a mean age of 38.0±6.7 years. Seminal samples were first analyzed in their fresh state and subsequently subjected to two cryopreservation protocols: (1) pre-refrigeration followed by exposure to liquid nitrogen (LN₂) vapor, and (2) direct exposure to LN₂ vapor. After thawing, sperm vitality, motility, and DNA fragmentation were reassessed. These parameters were systematically monitored to evaluate the potential influence of each protocol on post-thaw sperm quality. The following results summarize the comparative analysis of the two methods with respect to sperm motility and DNA integrity.

### Baseline Seminal Parameters

The pre-freezing seminal parameters are presented in Table 1, including sperm volume, concentration, motility, vitality, and morphology. All values were within WHO reference ranges (WHO, 2021). The mean sperm concentration was 47.7±41.0×10^6^/mL, total motility was 76.9%±9.1, vitality 85.1%±7.1, and normal morphology 4.0%±0.6.

**Table 1:** Patients’ age and standard semen parameters (n=21). Values are means±standard deviations (SD).

	Mean±SD	WHO ref.^*^
Age (years)	38.0±6.8	
Volume (mL)	3.8±2.3	≥1.4 mL
Sperm concentration (10^6^/mL)	47.7±41.0	≥16
Total sperm count (million)	161.2±113.7	≥42
Progressive motility (%)	49.0%±13.8	≥30%
Total motility (%)	76.9%±9.1	≥42%
Vitality (%)	85.1%±7.1	≥54%
Morphology (%)	4.0%±0.6	≥4%
^*^10th percentile of a fertile population (WHO, 2021)		

### Comparison of Fresh and Post-thaw Sperm Quality

As shown in Table 2, vitality, progressive motility, and total motility decreased significantly after cryopreservation in both the Refrigerated and Vapor Groups compared with the Fresh Group. Vitality decreased from 85.1% in fresh samples to 64.7% in the Refrigerated Group (*p*≤0.001) and to 63.7% in the Vapor Group (*p*≤0.001). Similarly, progressive motility dropped from 49.0% (Fresh) to 30.4% (Refrigerated) (*p*≤0.001) and to 27.4% (Vapor) (*p*≤0.001). No significant difference was detected between the two cryopreservation protocols in terms of vitality or motility recovery (*p*=0.718 and *p*=0.341, respectively).

**Table 2:** Progressive motility, total motility, and vitality in the Fresh Group (n=21), Refrigerated Group (n=21) *versus* Vapor Group (n=21). Values are means±standard deviations (SD).^^*^^Test Used: Paired T-test

Semen parameters	Fresh Group (1)	Refrigerated Group (2)	Vapor Group (3)	p 1 vs. 2	p 1 vs. 3	p 2 vs. 3
Progressive Motility	49%±13.8	30.4%±8.3	27.4%±5.9	≤0.001	≤0.001	0.341
Total motility	76.9%±9.1	59.7%±9.7	58.4%±8.9	≤0.001	≤0.001	0.361
Vitality	85.1%±7.1	64.9%±9.7	64.1%±15	≤0.001	≤0.001	0.718

### Recovery Rates of Vitality and Motility

Table 3 summarizes the recovery rates, defined as the post-thaw value expressed as a percentage of its corresponding fresh value, for vitality, progressive motility, and total motility. Both protocols yielded comparable recovery values. Vitality recovery was 76.7% in the Refrigerated Group and 76.1% in the Vapor Group (*p*=0.790). Progressive motility recovery was 64.9% in the Refrigerated Group and 67.3% in the Vapor Group (*p*=0.354). These findings suggest that both methods are equally effective in maintaining motility and vitality after thawing.

**Table 3:** Recovery rates after thawing relative to the Fresh Group for progressive motility, total motility, and vitality in the Refrigerated Group (n=21) and Vapor Group (n=21). Values are presented as means±standard deviations. Recovery rate (%)=(Post-thaw value ÷ Fresh value)×100.

Recovery parameter (relative to Fresh Group)	Refrigerated Group	Vapor Group	Mean difference (95% CI)	p^^*^^
Progressive Motility	64.6%±23	67.3%±21.8	+2.7 (-6.1 to +11.5)	0.354
Total motility	78.3%±13.4	76.6%±12.8	-1.7 (-8.5 to +5.1)	0.925
Vitality	76.7%±12.6	76.1%±18.8	-0.6 (-9.2 to +8.0)	0.790

^^*^^Paired t-test *(two-tailed)*(multivariate comparison)

### DNA Fragmentation

Table 4 presents the comparative analysis of sperm DNA fragmentation index (DFI), progressive motility, and vitality among the Fresh, Refrigerated, and Vapor groups. As shown, DNA fragmentation increased significantly post-thaw compared with fresh samples in both groups. When comparing the two protocols, the Vapor Group showed a lower mean DFI post-thaw compared to the Refrigerated Group (35.9% *vs*. 39.6%, *p*=0.001). In the Refrigerated Group, DFI increase from 27.4% (fresh) to 39.6% (post-thaw), representing a 44.5% increase (*p*≤0.001). In the Vapor Group, DFI increased from 27.4% to 35.9%, a 31.0% increase (*p*≤0.001). These findings suggest that the Vapor Protocol is more effective at minimizing DNA fragmentation during cryopreservation, thereby offering an advantage in preserving sperm DNA integrity. Logistic regression analysis suggested that the Vapor Group was associated with a lower likelihood of sperm DNA damage (β=-0.33, OR=0.72, 95% CI: 0.58-0.90, *p*=0.001). Although reductions in progressive motility and vitality were observed after cryopreservation, these changes were not significantly associated with the cooling protocol (*p*=0.108 and *p*=0.531, respectively), indicating that both methods had a similar effect on post-thaw sperm motility and survival.

**Table 4:** Comparative results of sperm DNA fragmentation (DFI), motility, and vitality, with logistic regression analysis for different cryopreservation protocols.

Parameter	Fresh group (1)	Refrigerated group (2)	Vapor group (3)	p^*^ (1 vs. 2)	p^*^ (1 vs. 3)	p^*^ (2 vs. 3)	β^†^	OR‡	95% CI^§^	p
DFI (%)^||^	27.4±9.6	39.6±11.4	35.9±10.5	<0.001	<0.001	0.001	-0.33	0.72	0.58-0.90	0.001
Progressive motility (%)	49.0±13.8	30.4±8.3	27.4±5.9	<0.001	<0.001	0.341	-0.15	0.86	0.70-1.04	0.108
Vitality (%)	85.1±7.1	64.9±9.7	64.1±15.0	<0.001	<0.001	0.718	-0.05	0.95	0.82-1.09	0.531

* Paired t-test and logistic regression model used for comparison.

^†^ β=logistic regression coefficient

‡ OR=odds ratio

§ CI=confidence interval

|| DFI= DNA fragmentation index

## DISCUSSION

### Summary of Main Findings

The present study compared two cryopreservation protocols with distinct cooling curves to determine which method was associated with better preservation of sperm DNA integrity. The main finding was that direct exposure to liquid nitrogen (LN_2)_ vapor was associated with lower post-thaw DNA fragmentation than the protocol involving prior refrigeration. Specifically, the post-thaw DNA fragmentation index (DFI) was lower in the Vapor group (35.9%) compared with the Refrigerated group (39.6%), with a smaller relative increase in fragmentation (31.0% *vs*. 44.5%). These results are clinically relevant, as elevated sperm DNA fragmentation has been strongly associated with reduced blastocyst formation, implantation failure, and higher miscarriage rates (Li *et al*., 2019; Lourenço *et al*., 2023; Simon *et al*., 2011).

Temperature monitoring in our study provided additional mechanistic insight: refrigerated samples stabilized at approximately 3°C, whereas those directly exposed to nitrogen vapor reached -80°C within 10 minutes, crossing the “critical temperature range” (-15°C to -60°C) more rapidly. A faster transition through this range likely reduced cryo-injury, supporting the hypothesis that rapid cooling helps preserve sperm cell integrity.

### Mechanistic Explanation

Our findings are consistent with previous evidence that cryopreservation is associated with DNA damage, with fragmentation being particularly sensitive to the freezing process. Riva *et al*. (2018) similarly reported that exposure to nitrogen vapor was associated with better preservation of DNA integrity than protocols involving refrigeration, likely due to less extracellular ice crystal formation and reduced oxidative stress. Slower cooling, as in refrigeration, may promote ice crystal formation and reactive oxygen species (ROS) generation, both of which could compromise membrane stability and DNA integrity (Isachenko *et al*., 2004; Said *et al*., 2010).

Conversely, rapid cooling minimizes intracellular ice formation and osmotic stress, thereby protecting cell membranes and organelles. In addition, oxidative stress may explain the greater DNA fragmentation observed in the refrigerated group, as cooling to 4°C has been shown to trigger peaks in ROS production, whereas lower temperatures tend to suppress ROS generation (Wang *et al*., 1997).

### Comparison with Literature

Although both motility and vitality significantly decreased after freezing, no significant difference was observed between the two cooling protocols, consistent with prior studies (Esteves *et al*., 2000; Til *et al*., 2016; Vaz *et al*., 2018). The recovery rates for motility (~63%) in our study were higher than those reported by Bandularatne & Bongso (2002), who found rates below 50% (Bandularatne & Bongso, 2002).

Despite similarities in motility outcomes, our results agree with those of Riva *et al.* (2018) who suggested that faster cooling may better preserve DNA integrity. However, some studies have reported conflicting results (Isachenko *et al*., 2004; Tongdee *et al*., 2015), likely reflecting differences in cryoprotectants, freezing devices, thawing procedures, and sample positioning relative to vapor exposure.

### Study Limitations and Implications

The present study has some limitations. The relatively small sample size and single-center design may limit the generalizability of the findings. Nonetheless, this design ensured consistent handling and strict protocol adherence, thereby strengthening internal validity. Although the study achieved adequate statistical power (80%), clinical outcomes such as fertilization or pregnancy rates were not assessed, and thus, the biological significance of the observed DFI difference (~3-4%) remains to be determined.

Despite these limitations, the paired design (each sample serving as its own control) and the precise temperature monitoring represent significant methodological strengths. Overall, our findings suggest that direct LN₂ vapor exposure may better preserve sperm DNA integrity than slower cooling involving prior refrigeration. This insight could contribute to the refinement of cryopreservation protocols in assisted reproductive technologies, where minimizing DNA damage is crucial to improving fertilization, embryo development, and pregnancy outcomes.

## CONCLUSION

In summary, cryopreservation using direct exposure to liquid nitrogen vapor was associated with lower sperm DNA fragmentation compared with protocols that included prior refrigeration. These findings suggest that faster cooling may help preserve sperm DNA integrity, a critical determinant of reproductive success. Further multicenter studies are warranted to confirm these associations and refine cryopreservation protocols. Optimizing sperm freezing techniques to minimize DNA fragmentation could ultimately contribute to improved outcomes in assisted reproductive technologies.
